# Radial Distribution Function in a Two-Dimensional Core-Shoulder Particle System

**DOI:** 10.1021/acs.jpcb.6c00653

**Published:** 2026-08-10

**Authors:** Michael Wassermair, Gerhard Kahl, Andrew J. Archer, Roland Roth

**Affiliations:** † 148492 Institut für Theoretische Physik, TU Wien, Wiedner Hauptstraße 8-10, A-1040 Vienna, Austria; ‡ Department of Mathematical Sciences and Interdisciplinary Centre for Mathematical Modelling, 27259 Loughborough University, Loughborough LE11 3TU, United Kingdom; § 5156 Institute of Science and Technology Austria, A-3400 Klosterneuburg, Austria; ∥ Institute for Theoretical Physics, 9188 University of Tübingen, D-72076 Tübingen, Germany

## Abstract

An important quantity in liquid state theory is the radial distribution function *g*(*r*). It can be calculated within the framework of classical density functional theory in two very distinct ways. In the test-particle route, one fixes a single fluid particle, turning it into an external potential in which the inhomogeneous structure of the fluid is calculated by minimizing the functional. The second route to *g*(*r*) in density functional theory employs the Ornstein–Zernike equation and the pair direct correlation function, that can be obtained from the second functional derivatives of the excess (over the ideal gas) free energy functional. Since typically an approximate excess free energy functional is employed, the test-particle route, which requires only one functional derivative, is more accurate than the Ornstein–Zernike route. Here we study a two-dimensional core-shoulder particle system and find that in some circumstances the results from the Ornstein–Zernike route can be comparable in accuracy to the test-particle results for *r* > σ, the core diameter. We also examine in detail the asymptotic *r* → ∞ decay of *g*(*r*), finding a variety of possible decay wavelengths at different state points and state points where there is a crossover from one wavelength to a very different one. This behavior is a signature pointing to the rich phase behavior of the incipient solid phases.

## Graphical abstract

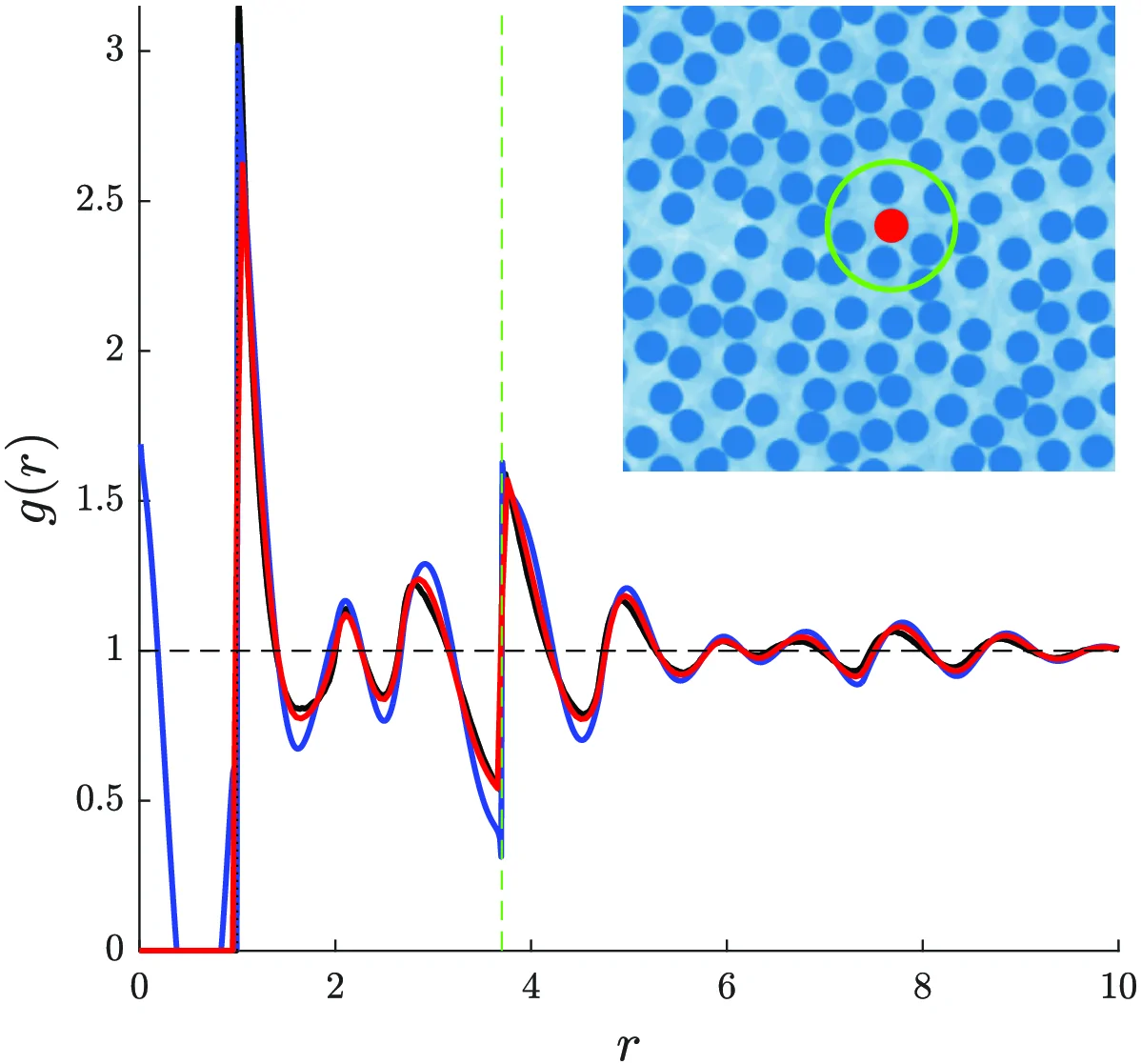

## I Introduction

The radial distribution function (RDF) *g*(*r*) of a simple fluid characterizes the local structure and correlations between particles and is an important quantity in liquid state theory.^1^ It establishes a bridge between the microscopic description of a fluid and macroscopic quantities such as the internal energy, the pressure or the static structure factor of the fluid (see, e.g., ref 1). The function *g*(*r*) measures the likelihood of finding a particle at distance *r* from a *test*-particle, relative to that in an ideal gas. For small separations *r* the structure of *g*(*r*) is strongly influenced by the particle–particle interaction. For fluids with a hard core of diameter σ = 2*R*, like the fluid we study here, *g*(*r*) displays a so-called correlation hole, which implies *g*(*r* ≤ σ) ≡ 0. The correlation hole is followed by the first correlation shell of roughly one particle diameter width, that contains information about the nearest neighbors of the test particle, followed by the second correlation shell and so on. Eventually for large distance *r* → ∞ particles are randomly placed relative to the test particle, like in the case of an ideal gas, and so as *r* → ∞, *g*(r) → 1. For short-ranged potentials, like the hard core shoulder potential employed in the present study, the decay of *g*(*r*) is exponential and the decay length is the correlation length.^1,2^ Nevertheless, the form of the decay of *g*(*r*) can be very informative and for the system we investigate here can exhibit a rich crossover behavior from oscillatory decay with one wavelength, to oscillatory decay with a very different wavelength. Understanding the origin of these different length-scales (wavelengths) is illuminating, because it gives hints toward the possible crystal structures that the system exhibits when the liquid freezes.^3,4^ Because of the importance of *g*(*r*) and the quantities that can be derived from it, there are many different approaches to compute this function, including classical density functional theory (DFT),^1,5,6^ integral equation theory that combines the Ornstein–Zernike (OZ) equation^7^ with a closure relation,^1^ and computer simulations.^8^

In this manuscript we employ DFT to study *g*(*r*) for a hard core square-shoulder fluid in two dimensions (2D) with an a radially symmetric interaction potential that is given by

1 $$\phi \left(r\right)=\{\begin{array}{ll} \infty & r\leq \sigma ,\\ \epsilon & \sigma <r<\lambda \sigma ,\\ 0 & \lambda \sigma \leq r \end{array}$$

where λσ is the range of the (repulsive) square-shoulder interaction with a height ϵ > 0. Within DFT one can prove^1,5,6^ that there exists the grand potential functional Ω­[ρ­(**r**)], that it is a functional of the ensemble averaged one-particle density ρ­(**r**), and that it is minimized by the equilibrium density distribution ρ_0_(**r**), for which the functional reduces to the grand potential Ω = Ω­[ρ_0_(**r**)] of the system. This can be written in the compact form of the Euler–Lagrange equation^6^

2 $${\frac{\delta \Omega \left[\rho \right]}{\delta \rho \left(r\right)}|}_{\rho \left(r\right)=\rho_{0}\left(r\right)}=0$$

Unfortunately, the mathematical proof of DFT, that guarantees the existence of the functional does not provide insight in how the functional, especially the part that describes interparticle interaction, is constructed. Several approximations for hard spheres in dimension *d* = 3 have been found,^9–11^ the most successful being fundamental measure theory (FMT).^11,12^ FMT follows the structure of the exact DFT for hard rods in *d* = 1,^13,14^ which makes use of the geometrical measures of the particle shape to account for the hard core repulsion. FMT functionals^11,15–18^ provide a very accurate description of hard sphere fluids compared to computer simulations. The ideas of FMT have been successfully transferred^19,20^ and applied to hard disk fluids in *d* = 2.^21^ Within the framework of DFT the soft (finite) part of the interparticle interaction in eq 1, is typically treated by a random phase approximation (RPA).^1,22^

DFT offers two very distinct routes to calculate *g*(*r*). The first approach is called the test-particle route and was suggested by Percus,^23,24^ who realized that a particle of the fluid can be fixed at e.g., the origin of our coordinate system and thereby be made an external potential acting on the rest of the fluid. The external potential *V*_ext_(*r*) = ϕ­(*r*) is the pair potential of the fluid, which in the present case is radially symmetric. If one minimizes the density functional to obtain the equilibrium density profile ρ­(*r*) in this external potential, it is related to the RDF via *g*(*r*) = ρ­(*r*)/ρ_b_, where ρ_b_ is the bulk density far away from the test particle. This approach requires one to only take one functional derivative of the *approximate* grand potential functional Ω­[ρ] in order to solve the Euler–Lagrange equation, eq 2, which can be done numerically using e.g., a Picard iteration.

The second approach makes use of the OZ relation, which connects the total correlation function *h*(*r*) = *g*(*r*) – 1 with the so-called pair direct correlation function (pDCF) *c* ^(2)^(*r*) via^1,7^

3 $$h\left(r\right)=c^{\left(2\right)}\left(r\right)+\rho_{b}\int c^{\left(2\right)}\left(|r-r^{\prime }|\right)h\left(r^{\prime }\right)dr^{\prime }$$

In order to solve this equation an additional closure relation which relates the functions *h*(*r*) and *c* ^(2)^(*r*) is required. However, within DFT it is possible to calculate the pDCF *c* ^(2)^(*r*) from the excess free energy functional *F*_ex_[ρ­(**r**)][Bibr ref6]

4 $$c^{\left(2\right)}\left(r=\left|r-r^{\prime }\right|\right)=-\beta \frac{\delta^{2}F_{\mathrm{ex}}\left[\rho \left(r\right)\right]}{\delta \rho \left(r\right)\delta \rho \left(r^{\prime }\right)}$$

which requires the second functional derivative of the *approximate* density functional. From a FMT functional it is possible to obtain a closed analytical expression for the pDCF, which can be employed to solve the OZ equation, i.e., the approximate excess free energy functional *F*_ex_[ρ­(**r**)] replaces the need for an additional (approximate) closure relation.

The core condition *g*(*r* ≤ σ) ≡ 0 for hard cores is often part of a closure relation,^1,25^ but is usually violated when a pDCF obtained via eq 4 is employed in the OZ equation.^26^ This is acceptable as long as the RDF outside of the core is accurate. Within DFT the typical experience is that *g*(*r*) predicted by the test-particle route is more accurate than the one obtained via the pDCF. The explanation or rationalization for this comes from the fact that a computation that requires only one functional derivative of an approximate functional should be more reliable than one that requires two functional derivatives. Another rationalization is the one discussed in ref 22, based on the observation that the test-particle Euler–Lagrange equation can be written in the form of the OZ equation, that turns out to have a hybrid closure relation that is better than one might expect from just considering eq 4. We discuss this issue in more detail in Appendix A of this paper. A somewhat surprising outcome of the present work is the observation that for *r* > σ the OZ result is almost as accurate as the test-particle result for sufficiently large values of the shoulder range λ. This is valuable, because the OZ expressions are analytic in Fourier space. This provides the basis for an analysis of the asymptotic decay behavior of *g*(*r*), which turns out to be very rich, exhibiting multiple crossovers in the phase diagram from damped oscillatory decay with one wavelength, to damped oscillatory decay with a very different wavelength.

In this manuscript we employ DFT for a two-dimensional (2D) hard core square-shoulder fluid^3,4^ in order to predict the RDF *g*(*r*) using the test-particle route and compare these results to *g*(*r*) obtained via the OZ equation together with a pDCF obtained from the same density functional. The manuscript is structured as follows. We present the theory in Section 2, where we specify the details of the density functional employed here, give details about the Monte-Carlo simulation approach used for obtaining benchmark *g*(*r*) data and other details relating to how we perform the calculations. In Section 3 we briefly discuss the theory for determining the *r* → ∞ asymptotic decay of *g*(*r*). In Section 4 we present our results. In Section 5 we make a few concluding remarks. In Appendix A we briefly discuss how the OZ equation in combination with the random phase approximation (RPA) closure relates to the RPA-DFT Euler–Lagrange equation in the test-particle limit, while in Appendix B we give a few of the technical details relating to our DFT calculations.

## II Theory

We consider a 2D one-component fluid with pair interactions between the particles, within the framework of DFT. The grand potential functional is given by^1,6^

5 $$\Omega \left[\rho \right]=F_{\mathrm{id}}\left[\rho \right]+F_{\mathrm{ex}}\left[\rho \right]+\int \rho \left(r\right)\left[V_{\mathrm{ext}}\left(r\right)-\mu \right]dr$$

where μ is the chemical potential and *V*_ext_(**r**) is the external potential. The ideal gas contribution to the free energy in two dimensions *F*_id_[ρ] is known exactly and is given by

6 $$F_{\mathrm{id}}\left[\rho \right]=k_{B}T\int \rho \left(r\right)\left[\ln\Lambda^{2}\rho \left(r\right)-1\right]dr$$

with the (irrelevant) thermal de Broglie wavelength Λ. The excess (over the ideal gas) Helmholtz free energy functional *F*_ex_[ρ], which contains all the information about the interparticle interaction, can be decomposed, within perturbation theory, into two contributions

7 $$F_{\mathrm{ex}}\left[\rho \right]=F_{c}\left[\rho \right]+F_{\mathrm{sh}}\left[\rho \right]$$

The first term incorporates the contribution due to the hard core particle interactions and represents the reference system for the fluid with the interaction given in eq 1. It is approximated within FMT,^3,11,12,20^ which makes the ansatz that the excess free energy functional is a volume integral of an excess free energy density, that is a function of position-dependent weighted densities. For a fluid of hard disks the related state of the art functional is given by^20^

8 $$F_{c}^{\mathrm{FMT}}\left[\rho \right]=k_{B}T\int \left[-n_{0}\left(r\right)\ln\left(1-n_{2}\left(r\right)\right)+\frac{1}{4\pi \left(1-n_{2}\left(r\right)\right)}\left(\frac{19}{12}{\left(n^{\left(0\right)}\left(r\right)\right)}^{2}-\frac{5}{12}n^{\left(1\right)}\left(r\right)\cdot n^{\left(1\right)}\left(r\right)-\frac{7}{6}n^{\left(2\right)}\left(r\right)\cdot n^{\left(2\right)}\left(r\right)\right)\right]dr$$

with weighted densities *n*_α_(**r**) (with α = 0, 2) and **n** ^(*m*)^(**r**) (with *m* = 0, 1, 2) that are convolutions of the local density ρ­(**r**) with geometrical weight functions ω_α_(**r**) and ω^(*m*)^(**r**). For hard disks one requires two scalar weighted densities

9 $$n_{\alpha }\left(r\right)=\left[\rho ⊗\omega_{\alpha }\right]\left(r\right),\alpha =0,2$$

and three tensorial weighted densities

10 $$n^{\left(m\right)}\left(r\right)=\left[\rho ⊗\omega^{\left(m\right)}\right]\left(r\right),m=0,1,2$$

The weight functions are^20^

11 $$\omega_{0}\left(r\right)=\frac{\delta \left(R-r\right)}{2\pi R}\;\mathrm{and}\;\omega_{2}\left(r\right)=\Theta \left(R-r\right)$$

where *R* = σ/2 is the radius of the disks, δ­(*r*) is the Dirac delta distribution and Θ­(*r*) is the Heaviside step-function. The tensorial weight functions are given by

12 $$\omega^{\left(m\right)}\left(r\right)=\delta \left(R-\left|r\right|\right)\underset{m‐\mathrm{times}}{\underset{︸}{\mathrm{r̂}...\mathrm{r̂}}}$$

The rank *m* tensorial weight function arises from taking *m* tensor products of the unit vector **r̂** with itself.

The second term in eq 7 is the following RPA approximation^1,3,6,22^

13 $$F_{\mathrm{sh}}\left[\rho \right]=\frac{1}{2}\int \int \rho \left(r\right)\rho \left(r^{\prime }\right)\phi_{\mathrm{sh}}\left(|r-r^{\prime }|\right)drdr^{\prime }$$

where

14 $$\phi_{\mathrm{sh}}\left(r\right)=\{\begin{array}{ll} \epsilon & 0<r<\lambda \sigma ,\\ 0 & \lambda \sigma \leq r \end{array}$$

is the repulsive shoulder part of the pair potential. Note that the repulsion has been extended inside the core of the particles.

Having fully specified the excess free energy functional, we are in the position to compute the pDCF using eq 4. Since the OZ equation, eq 3, can most easily be solved in Fourier space to give

15 $$ĥ\left(k\right)=\frac{{ĉ}^{\left(2\right)}\left(k\right)}{1-\rho {ĉ}^{\left(2\right)}\left(k\right)}$$

we require the Fourier transform of the pDCF *ĉ* ^(2)^(*k*). Note that the functional form of the excess free energy functional given in eq 7 implies that the pDCF, within perturbation theory, can be split into two terms, which obviously also holds for its Fourier transform

16 $${ĉ}^{\left(2\right)}\left(k\right)={ĉ}_{c}^{\left(2\right)}\left(k\right)+{ĉ}_{\mathrm{sh}}^{\left(2\right)}\left(k\right)$$

The first term, arising from the hard disk core repulsion treated using eq 8, makes use of the structure of FMT. One finds that the core contribution to the pDCF, eq 4 can be written as

17 $$c_{c}^{\left(2\right)}\left(r=\left|r_{1}-r_{2}\right|\right)=-\sum_{\alpha ,\gamma }\frac{\partial^{2}\Phi }{\partial n_{\alpha }\partial n_{\gamma }}\int dr^{\prime }\omega_{\alpha }\left(r^{\prime }-r_{1}\right)\omega_{\gamma }\left(r^{\prime }-r_{2}\right)$$

where Φ is the integrand in eq 8. Equation 17 can be transformed into Fourier space with the help of the convolution theorem, giving

18 $${ĉ}_{c}^{\left(2\right)}\left(k\right)=-\sum_{\alpha ,\gamma }\frac{\partial^{2}\Phi }{\partial n_{\alpha }\partial n_{\gamma }}{\mathrm{\omega ̂}}_{\alpha }\left(k\right){\mathrm{\omega ̂}}_{\gamma }\left(-k\right)$$

The second derivatives of Φ w.r.t. the weighted densities and the Fourier transforms of the weight functions are known analytically and hence we arrive at the following closed expression for *ĉ*_c_^(2)^(*k*)^3,21^

19 $${ĉ}_{c}^{\left(2\right)}\left(k\right)=\frac{\pi }{6{\left(1-\eta \right)}^{3}k^{2}}\left[-\frac{5}{4}{\left(1-\eta \right)}^{2}k^{2}J_{0}{\left(k/2\right)}^{2}+\left(4\left(\left(\eta -20\right)\eta +7\right)+\frac{5}{4}{\left(1-\eta \right)}^{2}k^{2}\right)J_{1}{\left(k/2\right)}^{2}+2\left(\eta -13\right)\left(1-\eta \right)kJ_{1}\left(k/2\right)J_{0}\left(k/2\right)\right]$$

where *J*_*n*_(*x*) are Bessel functions of order *n*. The second term in eq 16 is the contribution from the shoulder to the pDCF, generated by the functional in eq 13, and may be written as^3^

20 $${ĉ}_{\mathrm{sh}}^{\left(2\right)}\left(k\right)=-\beta 2\pi \epsilon \lambda \frac{J_{1}\left(\lambda k\right)}{k}$$

With the explicit expression for *ĉ* ^(2)^(*k*), the total correlation function *h*(*r*) can be calculated via an inverse Fourier transform.

As benchmark data for our DFT results we perform grand canonical Monte-Carlo (GCMC) simulation of the square-shoulder system, to obtain *g*(*r*). The GCMC simulations for the liquids were performed in square boxes of size 125σ × 125σ. A total of 20 × 10^9^ GCMC-moves was performed, where in each step particle translation, creation and deletion was attempted with equal probability $\alpha_{t}=\alpha_{c}=\alpha_{d}=\frac{1}{3}$ . For the densities considered the simulations contained roughly 2000–8000 particles. The RDF is calculated from the positions of the particles, **r**_*i*_ via^1^

21 $$g\left(r\right)=\frac{1}{\rho_{b}}\left\langle \frac{1}{N}\sum_{i=1}^{N}\sum_{j=1,j\neq i}^{N}\delta \left(r+r_{i}-r_{j}\right)\right\rangle$$

where the brackets denote a grand-canonical average. This ensemble average was performed over 250 configurations, which were separated by 20 × 10^6^ MC-moves along a Markov-chain.

## III Asymptotic Decay of Correlations

The properties of *g*(*r*) at intermediate values of *r* > σ reveals much about the local packing environment of the particles in a fluid. However, significant understanding of the structure in a fluid can also be understood from inspecting the *r* → ∞ decay behavior of *g*(*r*). The general theory for the decay of *g*(*r*) was developed by Evans and co-workers, initially for three-dimensional (3D) fluids^2,27,28^ and then more recently for 2D fluids.^29^ The starting point for the analysis is eq 15. Taking the inverse Fourier transform in 2D we obtain

22 $$h\left(r\right)=\frac{1}{2\pi }\int_{0}^{\infty }d\mathrm{kk}J_{0}\left(\mathrm{kr}\right)\frac{{ĉ}^{\left(2\right)}\left(k\right)}{1-\rho {\mathrm{b̂}}^{\left(2\right)}\left(k\right)}$$

where *J*_0_ is the zeroth Bessel function of the first kind. In 3D there is an equivalent but somewhat simpler formula that involves an exponential, rather than a Bessel function.^2,27,28^ The key idea is to evaluate this integral as a contour integral in the complex plane. The contour chosen is typically a semicircle in the upper half of the complex plane,^2,27–29^ but other choices are sometime possible.^30^ Evaluating the integral (eq 22) in this manner transforms it into a sum over residues of the poles of the integrand in the upper half of the complex plane. However, as we show below, the pole structure in the upper half of the complex plane is mirrored in the lower half, so evaluating via a contour around the lower half plane, is equally possible. The poles arise at points in the complex-*q* plane where the denominator in the integrand of eq 22 is zero, i.e., where

23 $$\left[1-\rho {\mathrm{b̂}}^{\left(2\right)}\left(q\right)\right]=0$$

Note that henceforth we denote complex wavenumbers with the letter *q*, while we denote real wavenumbers with the letter *k*. Typically, there are very many (possibly an infinite number) of poles, i.e., roots of eq 23. However, it is the pole(s) q_*n*_ = *k*_*r*_ + *ik*_*i*_ with smallest imaginary part *k*_*i*_ which determines the asymptotic decay of *h*(*r*), since each pole (together with its complex conjugate pair) contributes a term ∼ exp­(*iq*_*n*_ *r*) to *h*(*r*).^2,27–29^ If the pole is purely imaginary, then the asymptotic *r* → ∞ decay of *h*(*r*) takes the form

24 $$h\left(r\right)=\frac{A_{n}\exp\left(-k_{i}r\right)}{\sqrt{r}}+f$$

where *f* denotes terms having a *faster* decay, as *r* → ∞. A complex pole together with its conjugate pair, instead lead to a decay of the form^29^

25 $$h\left(r\right)=\frac{A_{n}\exp\left(-k_{i}r\right)\cos\left(k_{r}r+\theta \right)}{\sqrt{r}}+f$$

where θ is a phase shift.^2,27–29^ There are very similar results in 3D, except in 3D the √*r* in the denominator is replaced just by *r*.^2,27,28^ When there are two pairs of poles (i.e., four poles in total) that have the same value for their imaginary part *k*_*i*_, then there is a crossover from oscillatory decay with one wavelength, to oscillatory decay with another wavelength as one moves through that point in the phase diagram. Only a few one-component systems are known to exhibit such a crossover.^29,31^ It is much more common in binary mixtures, where there is often a crossover in the decay of the correlation functions *g*_*ij*_(*r*) as the relative concentrations of the two species *i* and *j* are varied, as long as there is a sufficient size difference between the particle size of the two species.^32–36^ Note also that there can be a crossover from monotonic decay to damped oscillatory decay. The line in the phase diagram at which this occurs is known as the Fisher-Widom line.^27,37^

The poles (and therefore the asymptotic decay) can be determined by solving eq 23 for complex *q*. A related quantity is

26 $$S\left(q\right)=\frac{1}{1-\rho {\mathrm{b̂}}^{\left(2\right)}\left(q\right)}$$

which when evaluated for *real q* = *k*_*r*_ = *k* yields the static structure factor *S*(*k*).^1^ Below we present results for the locations of the poles *q*_*n*_ and on the same plots display arg *S*(*q*), which is also illuminating.

As well as the static structure factor *S*(*k*), another related and physically relevant quantity is the dispersion relation^4,38,39^

27 $$\omega \left(k\right)=-Dk^{2}\left[1-\rho {\mathrm{b̂}}^{\left(2\right)}\left(k\right)\right]$$

where *D* is the diffusion coefficient. The dispersion relation determines the linear (small amplitude) growth or decay of density fluctuations in the uniform liquid. When one considers a small density perturbation of the liquid density of the form ρ­(**r**, *t*) = ρ + *δρ*(**r**, *t*), one finds that this evolves subsequently over time as the following Fourier sum

28 $$\delta \rho \left(r,t\right)=\sum_{k}{\mathrm{\rho ̂}}_{k}e^{ik\cdot r+\omega \left(k\right)t}$$

as long as the Fourier amplitudes ρ̂_**k**_ are small.

Since ω­(*k*) is a growth/decay rate, the wavenumbers *k* = *k*_*u*_ ≠ 0 where ω­(*k*) has a local maximum (i.e., where ∂ω­(*k*_*u*_)/∂*k* = 0) are physically relevant, since when ω­(*k*_*u*_) > 0, these are the fastest growing modes. The locus in the phase diagram where the largest local maximum in ω­(*k*) at *k* = *k*_*u*_^max^ has growth rate ω­(*k*_*u*_^max^) = 0 is referred to here as the linear stability threshold. To one side of this line in the phase diagram, we have ω­(*k*) < 0 for all *k* ≠ 0 and so the uniform liquid is linearly stable, while on the other side we have ω­(*k*) > 0 for *k* ≈ *k*_*u*_^max^ and so the liquid is unstable. This is where crystalline or quasicrystalline phases are to be expected.^4,39^ Note that this threshold is sometimes referred to as the λ-line.^40,41^ However, to avoid confusion with the shoulder range parameter, here we avoid referring to it this way. Owing to the close connections between ω­(*k*) and *S*(*q*)–see eq 26 and 27–there are close connections between the asymptotic decay of *h*(*r*) and shape of ω­(*k*), that we elucidate further below.

## IV Results

In the following we make use of some reduced units. We set the hard disk diameter σ = 1 as the unit of length and hence measure all other lengths in units of σ. The temperature *T* of the system sets an energy scale *k*_B_ *T*, where *k*_B_ is the Boltzmann constant. In the present system, the only other energy scale that can be compared to *k*_B_ *T* is the shoulder height ϵ in eq 1, which enters our calculation as the dimensionless quantity *β ϵ*, where β = 1/(*k*_B_ *T*), as usual. To change the temperature in our system we *effectively* set β ≡ 1 and use, following our previous convention,^3^ *T* = 1/ϵ. In this study our main interest is in the fluid phase. However, we do also present results for some quantities at state points where the solid phases arise in order to (i) show the contrast with properties in the liquid and (ii) to connect to the work in ref,^4^ on the solid phases.

We start by presenting results for *g*(*r*) for three different values of the interaction range, λ = 2.5, 3.7 and 4.9. These rather large values of the square-shoulder range give rise to interesting crystalline and quasicrystalline behavior at low temperatures.^4^ First, we discuss the RDF *g*(*r*) obtained using the test-particle approach (red curves in Figure 1). These are calculated by fixing one particle at the origin and turning it into an external potential for the rest of the system. The external potential is set to be equal to the pair potential, *V*_ext_(**r**) = ϕ­(*r*). It might seem best to make use of the radial symmetry of the problem and reduce the resulting DFT calculation to an effective one-dimensional (1D) problem. However, within FMT one reason for its good performance stems from the fact that the weighted densities are in **r**-space convolutions of the local density with geometrical weight functions, which can be evaluated fast and accurately using fast Fourier transforms (FFTs). If we reduce the dimensionality of the problem from 2D to effective 1D, weighted densities lose the property of being convolution products and the calculation becomes far less efficient and the computation time increases by orders of magnitude. For this reason we stay with a full 2D system to perform our calculations, using uniformly discretized Cartesian coordinates, which do not allow for an exact representation of the hard core of the external potential. This problem can be mitigated by averaging the resulting density profile ρ­(**r**) = ρ­(*r*, φ) over the angle φ to obtain

29 $$g\left(r\right)=\frac{1}{2\pi }\int_{0}^{2\pi }\frac{\rho \left(r,\varphi \right)}{\rho_{b}}d\varphi$$

where φ is the polar angle of **r**. A few further details regarding our DFT calculations are given in Appendix B. While there are alternative numerical approaches, that allow for a computationally efficient reduction of the 2D to an *effective* 1D problem like quasi-spectral methods^42^ or employing a logarithmic grid,^43,44^ we decided to stick to the 2D implementation, because the results presented here are part of a larger project.^3,4^

**Figure 1:**
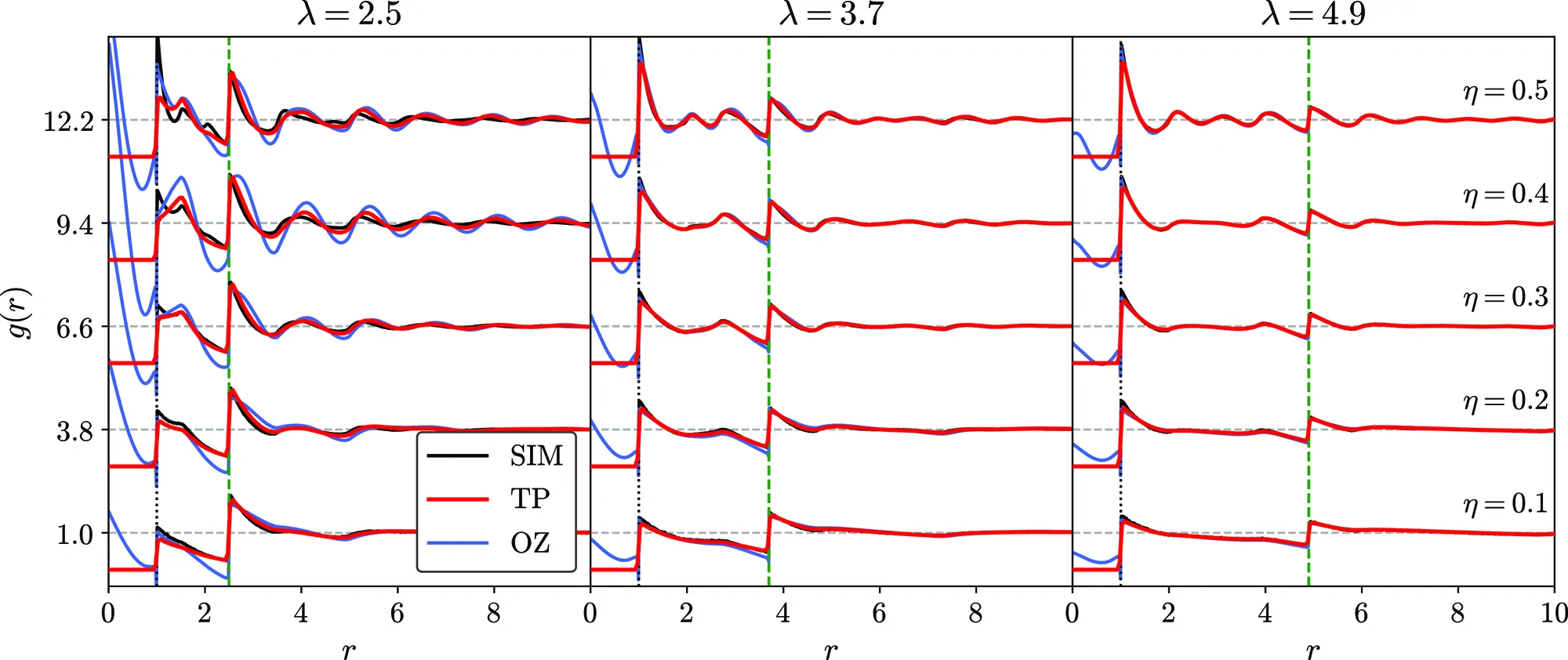
RDFs *g*(*r*) as functions of *r* for the core-shoulder fluid, calculated for three different values of the shoulder range λ (increasing from left to right, as labeled) and for five different values of packing fraction η (decreasing from top to bottom, as labeled). For better visibility, the curves are shifted vertically, with a 2.8 unit shift between each set of curves. The simulation (SIM) results are the black lines, the test-particle (TP) results are the red lines and data obtained via the Ornstein–Zernike (OZ) approach outlined in the text are the blue lines. The temperatures of the states are located at *T* = 1.25 × *T*_max_^λ^, where *T*_max_^λ^ are the temperatures of the maxima of the respective linear stability threshold lines. The corresponding temperature values are: *T* = 0.4748 (λ = 2.5), *T* = 0.8827 (λ = 3.7), and *T* = 1.4618 (λ = 4.9).

In Figure 1 we show our DFT result for the test-particle *g*(*r*), calculated using FMT (red lines) for five different values of the bulk packing fraction η = πρ_b_/4, where we have made use of σ = 1, for a fixed shoulder range λ = 2.5, 3.7 and 4.9. The calculations are performed at a fixed reduced temperature *T* = 1.25 × *T*_max_^λ^, where *T*_max_^λ^ is the temperature of the maxima of the linear stability threshold line^4^ for the given value of the shoulder range λ. The respective temperature values are *T* = 0.4748 for λ = 2.5, *T* = 0.8827 for λ = 3.7, and *T* = 1.4618 for λ = 4.9. Due to the hard core of the fixed test-particle at the origin, the RDF *g*(*r*) is enforced to vanish for *r* < 1. At *r* = 1 the RDF makes a jump from zero inside the core to its contact value. For *r* > 1 the RDF displays an oscillatory behavior due to packing effects. Note also that at *r* = λ the interaction potential ϕ­(*r*) and hence the external potential in the test-particle geometry jumps from ϵ > 0 to zero and as a result the RDF also changes discontinuously at *r* = λ.

We find that *g*(*r*) displays a complex, oscillatory behavior, which becomes more pronounced as the packing fraction η increases. This feature is well captured by the test-particle results (red lines), which is not too surprising because FMT accounts for short ranged correlations due to hard cores. In order to test the quality of the test-particle results within DFT we compare them to GCMC data, which are shown as black lines in Figure 1. We find that the overall behavior of the simulation-based *g*(*r*) is captured well by the test-particle results, albeit with contact values of *g*(*r*) and its values at the square shoulder distance *r* = λ a little too small. The accuracy of the test-particle results improve with increasing shoulder range λ. It is worth mentioning that the structure of *g*(*r*) for hard disks and for hard disk mixtures can be accounted for by FMT test-particle with much higher precision^20,21^ compared to the results presented in Figure 1.

As already mentioned, a second approach to *g*(*r*) within DFT makes use of the OZ eq 3. Instead of a closure relation, we employ the pDCF from DFT, derived from the excess free energy functional via eq 4. It is important to realize that for hard disks the OZ route to *g*(*r*) is typically significantly less accurate than the test-particle route, while it still predicts reasonable results for the pDCF obtained from FMT. The results from this OZ route are displayed in Figure 1 as blue lines. The first observation we can make is that the core condition *g*(*r* < 1) = 0 is violated by the OZ route. This observation has been made before for the OZ route using a pDCF obtained from an approximate excess free-energy functional, see e.g., ref 26. In order to avoid such a deficiency, closure relations like the Percus–Yevick closure,^1,25^ enforce the core condition. However, we also observe in Figure 1 that outside the core, the OZ results show a reasonable agreement with the computer simulations (black lines). Furthermore, the OZ route results for larger λ, such as λ = 3.7 and 4.9, are comparable in accuracy to those from the test-particle route, agreeing outside of the core surprisingly well with our simulation results.

These observations are valuable, because the pDCF contribution due to the core can be calculated analytically within FMT in Fourier space, resulting in the expression for *ĉ*_c_^(2)^(*k*) in terms of Bessel functions given in eq 19. The shoulder contribution to the pDCF can also be calculated analytically in Fourier space and again contains a Bessel function; see eq 20. While in odd dimensions (*d* = 1, 3,...) the Fourier transforms of the FMT weight functions can be expressed with trigonometric functions, in even dimensions (*d* = 2,4,...) one finds Bessel functions instead. Once the pDCF in Fourier space is given, the OZ equation can be solved using eq 15 via an inverse Fourier transform. In *d* = 2 this results in an inverse Hankel transform.

Having shown that the OZ route results are rather accurate and therefore the results we have for *ĉ* ^(2)^(*k*) in eqs 16, 19 and 20 are also reliable, we can now use these to determine the pole structure in the complex *q* plane. In Figure 2 we display examples at two different temperatures of where the poles are located, for the square-shoulder system with λ = 3.7 and a (reservoir) packing fraction of η = 0.5. The two different temperatures correspond to ϵ = 1.0 on the left and ϵ = 3.0 on the right, respectively. The red circles indicate the locations of (complex) poles and the color of the background heatmap denotes the phase of the (complex-valued) structure factor, i.e., arg *S*(*q*).

**Figure 2:**
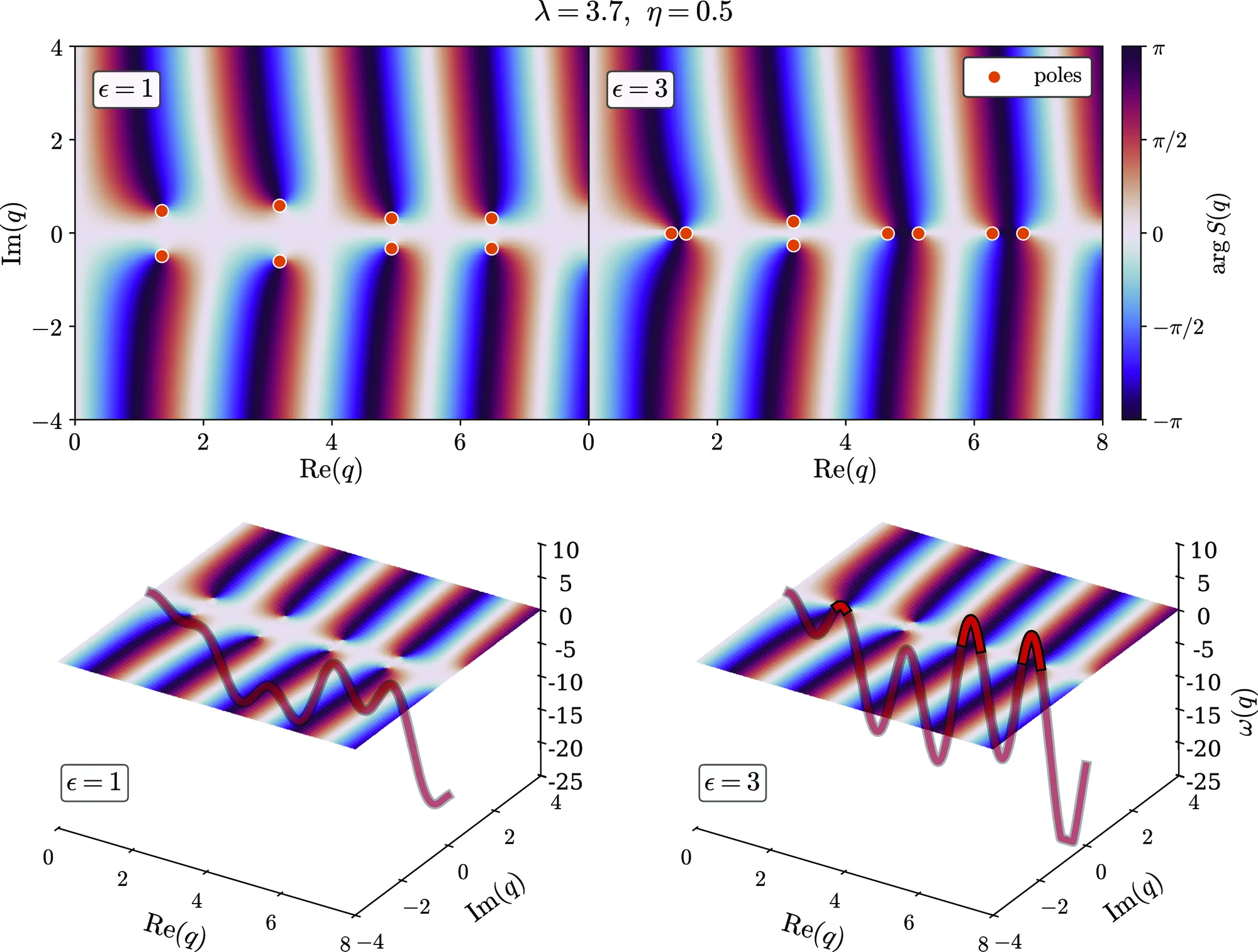
Top: Location of the poles of the complex-valued structure factor *S*(*q*) defined in eq 26 in the complex plane *q* = *k*_*r*_ + *ik*_*i*_ for a system with λ = 3.7, η = 0.5 and two different temperatures, ϵ = 1.0 (left panels) and ϵ = 3.0 (right panels). The positions of the poles, where 1/*S*(*q*) → 0, can be tracked by following the locations of the topological defects in the argument, arg *S*(*q*). Conjugate pairs of poles near the real axis give rise to peaks in the dispersion relation ω­(*k*) along the real axis, where *q* = *k* is real. In the lower plots, ω­(*k*) is displayed as the red line. When ω­(*k*) > 0, a density mode becomes unstable and the corresponding poles become real-valued; these are the zero-crossings of ω­(*k*). Hence, the position of the maxima *k*_*u*_ of ω­(*k*) lying between two consecutive poles along the real axis can be identified as wavenumbers corresponding to unstable density-modes.

The detection of poles of the complex-valued structure factor *S*(*q*) in eq 26 is realized by tracking “topological defects” in the phase ϕ = arg *S*(*q*). To this end we apply a simple, yet (with sufficient resolution) robust algorithm that is used in the context of vortex line tracking in random wave patterns.^45^ The poles are located by evaluating *S*(*q*) on a regular Cartesian grid with spacing Δ*q* = 0.013/σ and then identifying 2 × 2 neighboring grid points with consistent winding orientation. Thinking of this as an image analysis algorithm, then this corresponds to identifying properties of 2 × 2 pixel stencils. Thus, in the present context, we refer to *pixel* as a discrete lattice position in the complex plane and the 2 × 2 pixel stencil is formed by four lattice positions on a square. The algorithm consists of two steps:

(a) for each boundary between pixels *i* and *j* compute the phase difference and orient it into the direction that minimizes the absolute phase difference modulo 2π. This can be expressed as the principle argument △ϕ_*ij*_ = arg­(*e* ^*i*(ϕ_*j*_–ϕ_*i*_)^) where arg(·) ∈ [-π,π] or equivalently as $△\phi_{\mathrm{ij}}=\left(\phi_{j}-\phi_{i}\right)-2\pi \left\lfloor \frac{\phi_{j}-\phi_{i}+\pi }{2\pi }\right\rfloor$ .
(b) Within each 2 × 2 pixel stencil the four orientations of phase differences must have identical orientations when traversed in a (counter-)­clockwise direction for a topological defect to be present.

The sum of the four discrete phase differences approximates the closed contour integral of the winding number $W=\frac{1}{2\pi }\oint_{C}\nabla \phi \left(r\right)dl$ , which in our case is ±1.

In the fluid phase, the poles are complex with nonzero imaginary part and for ϵ ⩾ 0 appear in pairs of complex conjugates, as can be seen on the left-hand side of Figure 2. To determine *h*(*r*) for all *r*, one must sum over contributions from all of the displayed poles, as well as the (in principle) infinite number of poles in the upper half of the complex *q* plane that are not displayed. However, the large-*r* asymptotics of *g*(*r*) is dominated by just the leading order pole, which possesses the smallest imaginary part, because this pole corresponds to the slowest decay as given in eq 25. Additionally, the real part of the pole determines the wavelength of oscillation in the asymptotic decay, i.e., determining the dominating length scale in the pair correlation.

Some additional insight into the form of the structure factor *S*(*q*) in eq 26 and the dispersion relation ω­(*k*) in eq 27 can come from inspecting Figure 2. While *S*(*q*) (and so also ω­(*k*)) is a real function on the real axis, they manifest signatures of the close complex poles in form of maxima. In the lower panel of Figure 2 we show the structure of the dispersion relation ω­(*k*) in relation to the complex pole structure. Close by poles result in a local maximum in the dispersion relation. In the fluid phase, the dispersion relation ω­(*k*) remains negative for all *k* > 0 and modes with a given wavenumber *k* decay over time – see eq 28.

Where the uniform liquid is linearly unstable (i.e., where solid phases form), we have ω­(*k*) > 0 for some *k* > 0.^4,39^ This is illustrated in the right-hand panels of Figure 2, where we display the pole structure (top panel) and the corresponding dispersion relation (lower panel) for a state with the same packing fraction η = 0.5 and shoulder range λ = 3.7 as on the left-hand side of Figure 2, but at a significantly lower temperature, corresponding to ϵ = 3.0. At this state point, there exist poles on the real axis, which imply portions of the dispersion relation being positive. This pole structure, how they are located and the corresponding dispersion relation, which contains positive regions, is of interest for finding complicated solid structures^3,4^ since these define the wavenumbers of modes that can grow if the uniform liquid is quenched to such a state point.

In Figure 3 we display the asymptotic decay behavior of the total correlation function *h*(*r*) = *g*(*r*) – 1 for three different values of λ and several values of the packing fraction η. The first way of highlighting the asymptotics is by plotting the logarithm of *h*(*r*) multiplied by √*r*. This functional form is a consequence of the leading order expansion of the total correlation function in eq 25, that makes use of the pole of the structure factor with the smallest imaginary part. In the upper panel we compare the full solution from the OZ route to *h*(*r*) (full black lines) with those obtained from the leading order pole (broken colored lines). The packing fraction η increases from bottom to top between 0.1 and 0.6 in increments of 0.1. For reasons of clarity the plots for different values of η have been shifted vertically. We find overall very good agreement between the two results for both large and intermediate values of *r* > λ. Some numerical problems with the OZ results at low packing fractions and large *r* can be seen, but the reason is easily understood by observing fast decaying correlations in those cases.

**Figure 3:**
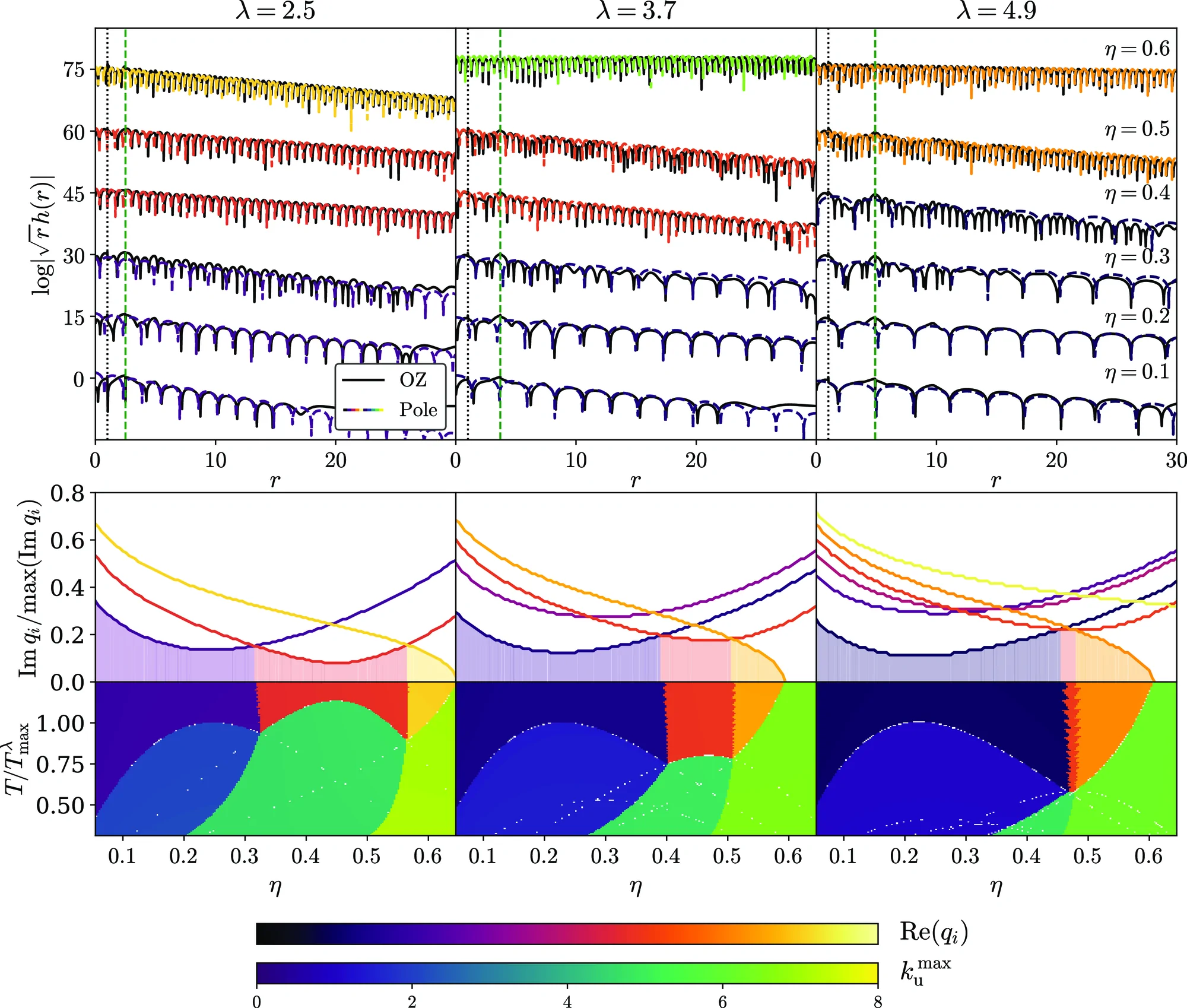
Top panels: plots of log­[√*rh*(*r*)], where *h*(*r*) = *g*(*r*) – 1, as functions of distance *r*, obtained via the OZ-route (solid black lines), compared to their asymptotic behavior in eq 25 (broken colored lines) obtained via the pole *q*_*n*_ = *k*_*r*_ + *ik*_*i*_ with the smallest imaginary part min_*i*_(Im*q*_*i*_). These functions are calculated for three different values of shoulder range λ (increasing from left to right, as labeled) and six different values of packing fraction η (decreasing from top to bottom, as labeled). For better visibility the curves are shifted vertically by 15 units. Middle panels: imaginary parts of the poles Im*q*_*i*_ (normalized via the respective maximum values), as functions of η, with the same λ-values as in the corresponding panels above. We have also used the same temperatures as in Figure 1, with varying packing fractions η. The shaded areas below the curves (colored with the same colors as the respective curves) indicate the η-interval where each pole *q*_*i*_ respectively assumes the minimum value. Lower panels: phase diagrams displaying in each case the corresponding linear stability threshold. The coloring above indicates the wavenumber of the (oscillatory) decay of *h*(*r*) and the coloring below giving the value of *k*_*u*_^max^, the most unstable (fastest growing) wavenumber obtained from ω­(*k*).

An interesting observation to be made from Figure 3 (top panels) is that the wavelength of the oscillations in *h*(*r*) changes (several times in some cases, depending on λ) from a large wavelength at low packing fractions to a smaller wavelength at high packing fractions. While such a behavior can occur continuously, here it happens rather sharply at intermediate values of the packing fraction η. Such a crossover has been predicted previously in a binary mixture of hard spheres (in *d* = 3) based on the pole analysis of the total correlation function^33,34^ and was called structural-crossover. The prediction was later confirmed experimentally in effective 2D colloidal mixtures^35^ and in 3D.^36^ For a one-component square-shoulder fluid in *d* = 1 structural crossover was also reported.^46^

In the middle panels of Figure 3 we demonstrate that the observed crossover occurs as a result of a competition between poles with different real parts, that correspond to different wavelengths. For the system with λ = 2.5 we plot the imaginary part of the three leading order poles, each having different real parts. At low packing fraction η, the purple line corresponds to the leading order pole. This pole has the smallest real part *Re*(*q*) and so the longest wavelength oscillatory decay contribution to *h*(*r*). At η ≈ 0.3 the pole corresponding to the red line becomes the leading order pole. Note that close to the transition both poles contribute to the asymptotic decay, because both poles contribute a term with similar exponential decay. Finally at η ≈ 0.55 the pole corresponding to the yellow line displays the slowest decay. Out of the three poles, this pole has the largest real part *Re*(*q*) and so the shortest wavelength oscillatory decay contribution to *h*(*r*). For even larger values of η the leading order pole approaches the real axes, indicating an instability of the fluid phase.

In the bottom panels of Figure 3 we sketch in each case the phase diagram as a function of η and *T*, showing the linear stability threshold line. In the linearly stable uniform liquid above this threshold line (defined above), the leading order pole determines the correlation length and the main wavelength of oscillation, the value of which is given by the background coloring. At lower temperatures, in the regions below the linear stability threshold lines, the background coloring indicates the value of *k*_*u*_^max^, the fastest growing wavenumber, corresponding to the largest maximum of ω­(*k*). This plays an important role in determining a characteristic lengthscale in the solid phases that arise in this region of the phase diagram.^4^

As we increase the value of λ, we see in Figure 3 that the situation becomes slightly more complicated. For λ = 3.7 we have to consider the four lowest lying poles in order to capture the behavior of *h*(*r*) in the fluid phase over the full η range. At sufficiently high values of η, the leading order pole again tends toward the real axes and the corresponding total correlation function *h*(*r*) from the OZ route for η = 0.6 (top panel) does not display a decay. In the corresponding middle panel, we see the values of Im­(*q*_*i*_) for the four leading order poles and in the bottom middle panel, the corresponding phase diagram displays the close connection to the pole structure of the uniform fluid phase. For the largest value of λ = 4.9, considered here, we plot the imaginary parts of the six lowest poles, which allows one rationalize the oscillatory structure of the total correlation function *h*(*r*) and the overall shape of the phase diagram.

In Figure 4, we again display the phase diagram and the linear stability threshold line, but this time we color the region above it according to the value of min_*i*_ Im­(*q*_*i*_), which is proportional to the reciprocal of the bulk fluid correlation length, i.e., the decay length of *h*(*r*). The bulk correlation length diverges on approaching the linear stability threshold, so we see that on approaching the linear stability threshold from above, min_*i*_Im­(*q*_*i*_) → 0. Below the linear stability threshold, we color the background to indicate the fastest growth rate from the dispersion relation. The fastest growth rate is given by the maximum of ω­(*k*), which we denote max_*i*_ ω_*i*_. In order to have a value on a similar scale to the quantity displayed above the linear stability threshold, we instead plot the quantity log­(1+ max_*i*_ ω_*i*_). We see that the growth rate increases with decreasing temperature, moving down from the linear stability threshold, and that the largest growth rates are to be found at low temperatures and higher densities, i.e., deep in the region where the solid phases are to be found.^4^

**Figure 4:**
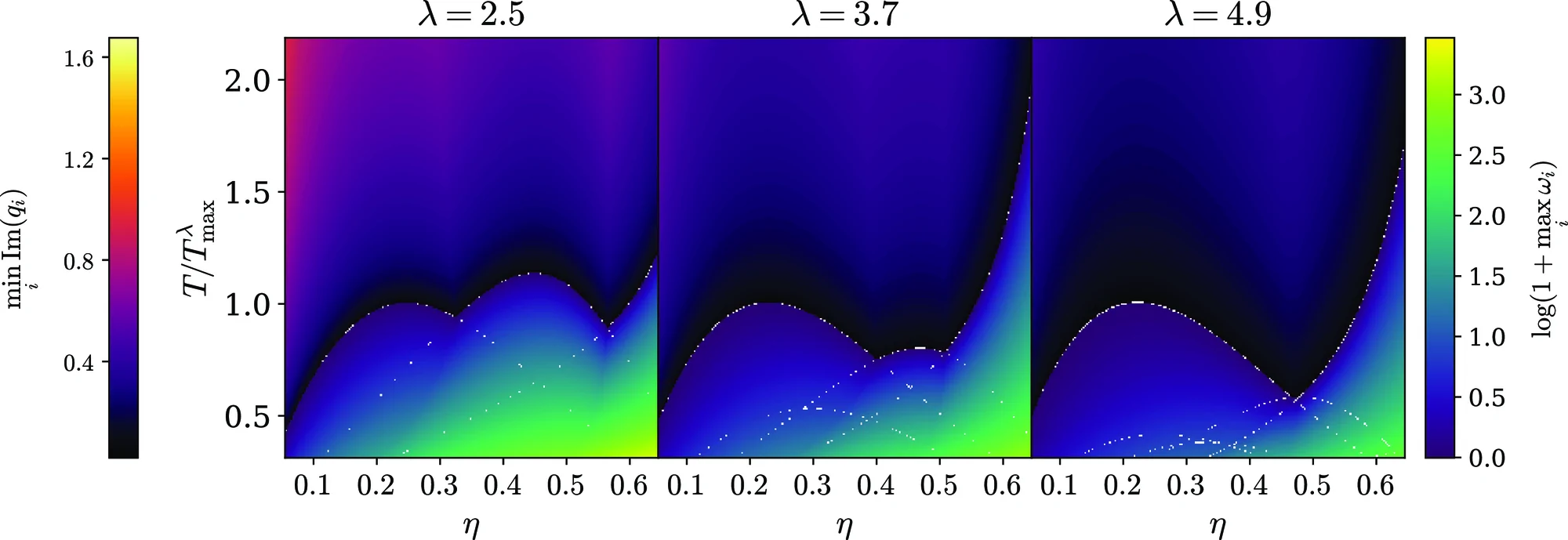
Repeat of the phase diagrams from along the bottom of Figure 3, showing the linear stability threshold lines for three values of λ. Here we instead color in each case the region above the line according to the value of the reciprocal of the bulk fluid correlation length, min_*i*_Im­(*q*_*i*_), and below the line with the value of log­(1 + max_*i*_ ω_*i*_), so as the indicate the regions where the growth rate max ω­(*k*) = max_*i*_ ω_*i*_ is largest.

### IV.I Poles for Small and Negative ϵ

We now conclude our results section by briefly presenting plots showing where the poles of the structure factor *S*(*q*) lie in the complex-*q* plane (and where they move to) in two different particular cases.

The first case we consider, displayed in Figure 5, illustrates the behavior in the limit ϵ → 0, i.e., when the pair potential shoulder-height tends to zero. These results are for η = 0.4, λ = 3.7 and the six different values of ϵ = 0, 0.001, 0.01, 0.1, 0.2 and 0.4. The value λ = 3.7 is the same as for the results displayed in Figure 2, but Figure 5 is for a somewhat lower density. Comparing the six sets of results in Figure 5, we go from the case ϵ = 0, which is just a pure hard-disk fluid, to cases with more substantial values of ϵ, where the square-shoulder contribution is much more significant. For ϵ exactly equal to zero, we have many fewer poles (only four in the portion of the complex-*q* displayed). For ϵ > 0, many more poles are introduced and we see that as ϵ is increased, these poles move toward the real axis, so that several of these poles have a comparable value of Im­(*q*) and so make a contribution to the decay of *h*(*r*) and the structure of the liquid. This illustrates how the presence of the shoulder in the pair potential introduces additional lengthscales into the liquid structure and correlations.

**Figure 5:**
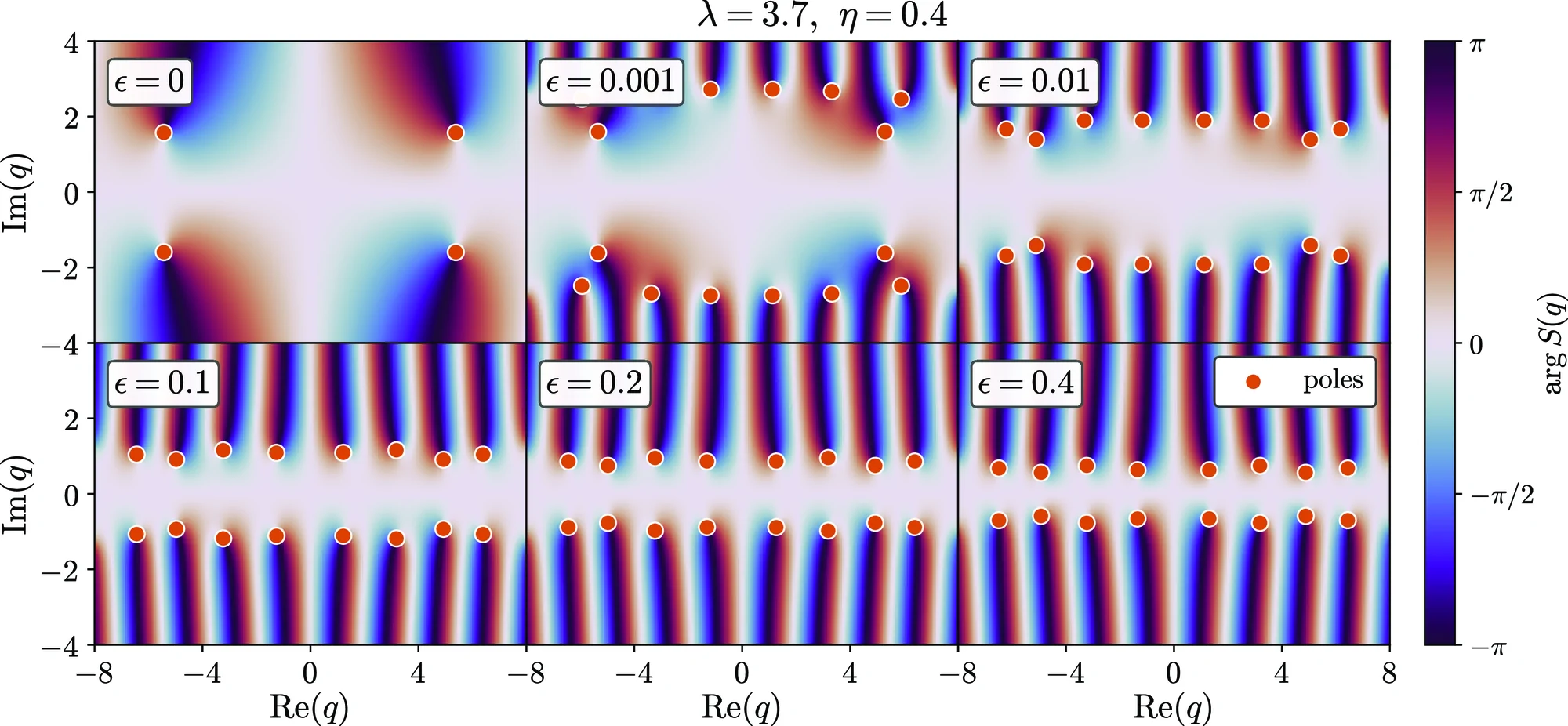
Plots of the locations of the poles of *S*(*q*) in the complex *q*-plane for λ = 3.7, η = 0.4 and varying ϵ, as indicated above. In the limit ϵ → 0, the system becomes a pure hard-disk fluid. The background coloring shows the value of the phase, arg *S*(*q*). This should be compared with the plots at the top of Figure 2, which are similar, but are for a higher value of η = 0.5 and for the cases ϵ = 1.0 and 3.0.

The second case we consider, displayed in Figure 6, shows the behavior when ϵ is negative, i.e., when the pair potential becomes *attractive*. Except for the sign of the chosen values of ϵ, the parameter values used in Figure 6 are exactly the same as those used in Figure 5. At face value, the plots in Figure 6 look somewhat similar to the corresponding plots in Figure 5, which is somewhat surprising, since an attractive shoulder is very different to a repulsive one. On closer inspection, the main key difference is the appearance of a *purely imaginary* pole for negative ϵ. A purely imaginary pole leads to a monotonically decaying contribution to *h*(*r*). As ϵ becomes increasingly negative, this pole moves down the imaginary axis and eventually near ϵ ≈ −0.3 it hits the real axis. This corresponds to meeting the spinodal associated with liquid–gas phase separation.^27^ In contrast, for ϵ > 0 we see no sign of this purely imaginary pole.

**Figure 6:**
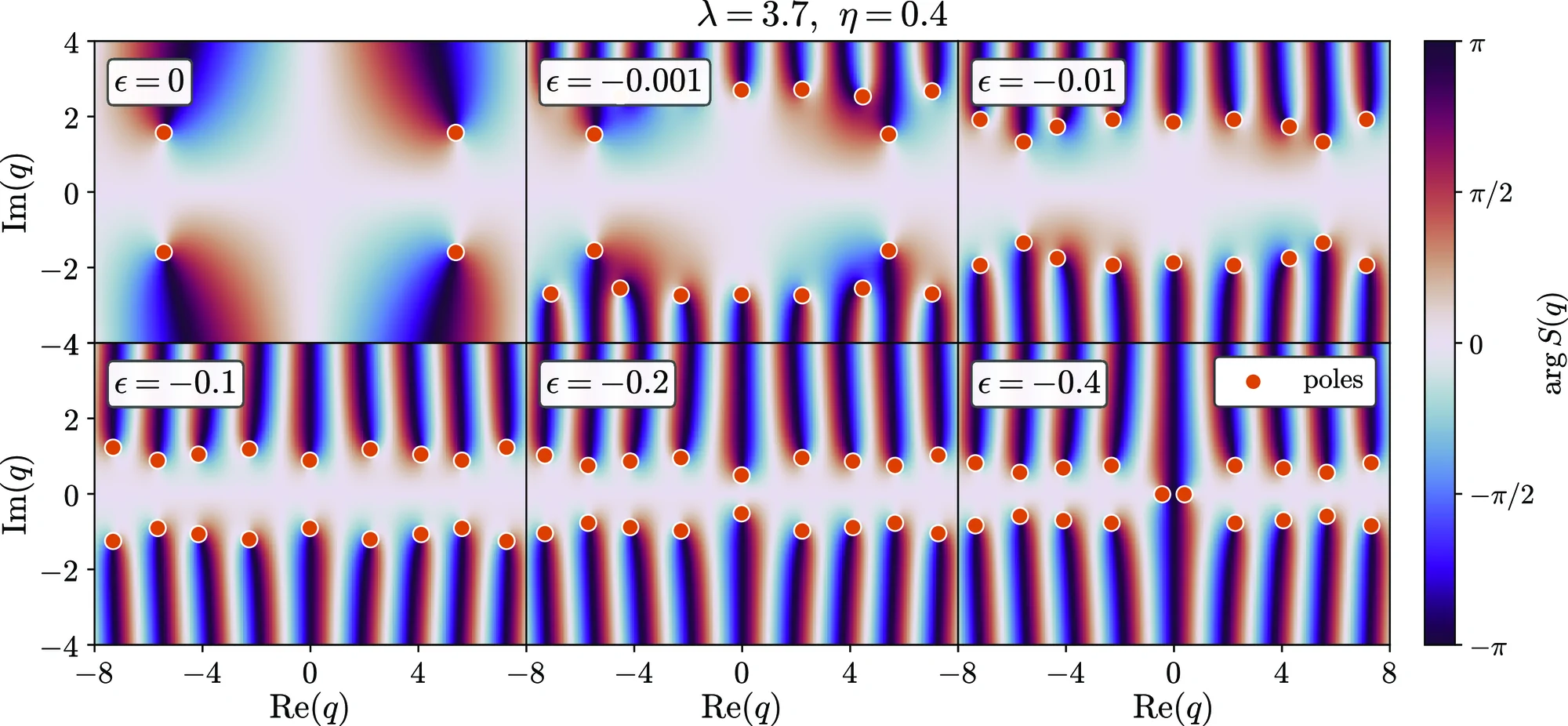
This plot is the same as Figure 5, except here the values of ϵ are *negative*, corresponding to an *attractive* square-well fluid.

## V Discussion and Summary

In this study we have considered a 2D system where the particles interact via a hard core interaction plus an adjacent square-shoulder potential whose range λ is rather long, notably up to 4.9 times the hard core diameter. We have calculated the RDF *g*(*r*) for the system via different routes. Within the DFT approach we have used the highly successful FMT for the core contributions and treated the shoulder via a standard perturbation theory approach, employing the RPA. We find that the DFT OZ route to *g*(*r*) can be of comparable accuracy to the generally more reliable test-particle approach, particularly for *r* > σ, outside of the core. For this reason, we have been able to apply with confidence our analytic OZ DFT results for the structure factor *S*(*q*) and to determine the locations and distribution of the poles of *S*(*q*) in the complex-*q* plane. These poles determine the asymptotic decay behavior of the total correlation function *h*(*r*) and we have found a very rich crossover behavior in its decay as η is varied, depending also on the particular value of λ. Moreover, we have also been able to elucidate much about the connections between the properties of these poles and the form of the dispersion relation ω­(*k*), which was shown to be remarkably useful for predicting properties of the crystalline and quasicrystaline phases exhibited by the present system.^4^

Of course, the DFT we have used is not exact and so could in principle be improved upon. We are confident that the main shortcoming of our DFT approach originates from the simple perturbative approach for the soft repulsive square-shoulder interaction, which we treat with the standard RPA ansatz, eq 13. This conclusion is based on the fact that for the bare hard core interaction, FMT is able to predict very accurate RDFs via the test-particle route.^20,21^ It should be mentioned that there are more sophisticated perturbation theories available^47–49^ to take into account a potential tail; however, their implementation for the system at hand is definitely beyond the scope of this manuscript. A first step to improve the level of agreement between DFT- and simulation results, at least for the simple square-shoulder potential, might be to make use of the freedom we have to modify the shoulder interaction ϕ_sh_(*r*) within the hard core region, without affecting the total interaction (which is anyhow infinite inside the core). Such an approach is known as the *optimized* random phase approximation,^50^ which could improve the performance of the test-particle route. Trying to improve the consistency between the OZ and the test-particle route to *g*(*r*) will surely be a strategy to improve the overall accuracy of the DFT. A step in this direction might be done either by setting ϕ_sh_(*r*) to zero inside the core or to replace it by a suitably designed function which is adjusted such that the agreement between the DFT and the simulation data is optimized. To some extent, the level of self-consistency could also be improved by adjusting the core contribution of the RPA term with the help of exact sum rules that make use of the RDF *g*(*r*) employing the test-particle route.^51,52^

The work of this manuscript has even broader implications, because having an accurate theory for *c* ^(2)^(*r*) is not just about getting *g*(*r*) correct. There are several other important quantities that depend on *c* ^(2)^(*r*), including the isothermal compressibility. The importance of the static structure factor and the dispersion relation is that these can be used to quickly and easily map out where in the phase diagram the solid phases possibly arise. Recall that the dispersion relation determines the growth/decay rate of periodic density modes in the uniform liquid^38^ and can be employed to predict with remarkable accuracy the structure of the solid phases that form when the liquid becomes unstable.^4^ In fact, by tuning the dispersion relation allows to identify pair potential parameters and state points where, for instance, quasicrystalline phases arise.^4^ We find it surprising that the DFT used here can semiquantitatively predict phase boundaries and the wavelengths of the density modulations that determine the crystalline and quasicrystalline structures formed by core-shoulder particle systems. We believe all of these observations will be useful to bear in mind in future studies aimed at developing improved DFTs for core-shoulder and other systems.

## Appendix A

We provide here further details about the OZ and test-particle routes for calculating *g*(*r*) = 1 + *h*(*r*). The following arguments lean heavily on related arguments put forward in ref 22.

### A OZ Equation with the RPA Closure

The exact closure relation to the OZ eq 3 is often written as^1^

30
$$c^{\left(2\right)}\left(r\right)=h\left(r\right)-\ln\left(h\left(r\right)+1\right)-\beta \phi \left(r\right)+B\left(r\right)$$

where *B*(*r*) is termed the bridge function; *B*(*r*) is in general not known exactly. The RPA-DFT approximation used here, given in eq 16 or eq 20, can be written as

31
$$c_{\mathrm{RPA}}^{\left(2\right)}\left(r\right)=c_{c}^{\left(2\right)}\left(r\right)-\beta \phi_{\mathrm{sh}}\left(r\right)$$

where *c*_c_^(2)^(*r*) is the pDCF for the (purely repulsive) reference hard disk fluid, given in eq 17. Plugging eq 31 into the OZ eq 3, we obtain

32
$$h\left(r\right)=c_{c}^{\left(2\right)}\left(r\right)-\beta \phi_{\mathrm{sh}}\left(r\right)+\rho_{b}\int dr^{\prime }h\left(r^{\prime }\right)c_{c}^{\left(2\right)}\left(|r-r^{\prime }|\right)-\rho_{b}\int dr^{\prime }h\left(r^{\prime }\right)\beta \phi_{\mathrm{sh}}\left(|r-r^{\prime }|\right)$$

We now move on to derive a corresponding expression via the test-particle route.

#### B The Percus Test-Particle Route with the RPA DFT

Percus showed^13,14^ that the RDF *g*(*r*) is related to the density profile ρ­(**r**) = ρ­(*r*) around a fixed particle (positioned in the origin) that exerts an external potential equal to the pair potential ϕ­(*r*) as: *g*(*r*) = *h*(*r*) + 1 = ρ­(*r*)/ρ_b_. Using DFT, ρ­(*r*) may be obtained via eq 2. So, minimizing ([Disp-formula eq5]) with *V*_ext_(**r**) = ϕ­(*r*), the resulting Euler–Lagrange equation reads

33
$$\frac{\delta \Omega \left[\rho \right]}{\delta \rho }=k_{B}T\ln\left[\Lambda^{2}\rho \left(r\right)\right]+\frac{\delta F_{c}\left[\rho \right]}{\delta \rho }+\int dr^{\prime }\rho \left(r^{\prime }\right)\phi_{\mathrm{sh}}\left(|r-r^{\prime }|\right)+\phi \left(r\right)-\mu =0$$

Far from the test particle, at *r* → ∞ the density ρ­(r) → ρ_b_ and ϕ­(*r*) = 0. In this limit, eq 33 gives

34
$$k_{B}T\ln\left[\Lambda^{2}\rho_{b}\right]+{\frac{\delta F_{c}\left[\rho \right]}{\delta \rho }|}_{\rho_{b}}+\rho_{b}\int dr\phi_{\mathrm{sh}}\left(r\right)-\mu =0$$

If we subtract eq 34 from eq 33, we obtain

35
$$0=k_{B}T\ln\left(\frac{\rho \left(r\right)}{\rho_{b}}\right)+\frac{\delta F_{c}\left[\rho \right]}{\delta \rho }-{\frac{\delta F_{c}\left[\rho \right]}{\delta \rho }|}_{\rho_{b}}+\int dr^{\prime }\left(\rho \left(r^{\prime }\right)-\rho_{b}\right)\phi_{\mathrm{sh}}\left(|r-r^{\prime }|\right)+\phi \left(r\right)$$

Multiplying through by (−β) and adding (ρ­(*r*) – ρ_b_)/ρ_b_ to both sides, together with making use of the following functional Taylor expansion about the bulk density

36
$$\frac{\delta F_{c}\left[\rho \right]}{\delta \rho }={\frac{\delta F_{c}\left[\rho \right]}{\delta \rho }|}_{\rho_{b}}+\int dr^{\prime }\left(\rho \left(r^{\prime }\right)-\rho_{b}\right){\frac{\delta^{2}F_{c}\left[\rho \right]}{\delta \rho \left(r\right)\delta \rho \left(r^{\prime }\right)}|}_{\rho_{b}}+H_{c}\left[\rho \left(r\right)\right]$$

where *H*_c_[ρ­(**r**)] denotes all higher order terms which are $∼O\left({\left[\rho -\rho_{b}\right]}^{2}\right)$ and higher, we obtain

37
$$\frac{\left(\rho \left(r\right)-\rho_{b}\right)}{\rho_{b}}=\frac{\left(\rho \left(r\right)-\rho_{b}\right)}{\rho_{b}}-\ln\left(\frac{\rho \left(r\right)}{\rho_{b}}\right)-\beta \phi \left(r\right)+\rho_{b}\int dr^{\prime }\frac{\left(\rho \left(r^{\prime }\right)-\rho_{b}\right)}{\rho_{b}}\left[{\frac{-\beta \delta^{2}F_{c}\left[\rho \right]}{\delta \rho \left(r\right)\delta \rho \left(r^{\prime }\right)}|}_{\rho_{b}}\right]-\beta H_{c}\left[\rho \left(r\right)\right]+\rho_{b}\int dr^{\prime }\frac{\left(\rho \left(r^{\prime }\right)-\rho_{b}\right)}{\rho_{b}}\phi_{\mathrm{sh}}\left(|r-r^{\prime }|\right)$$

Now, recalling eq 4, we therefore have that

38
$$c_{c}^{\left(2\right)}\left(|r-r^{\prime }|\right)={\frac{-\beta \delta^{2}F_{c}\left[\rho \right]}{\delta \rho \left(r\right)\delta \rho \left(r^{\prime }\right)}|}_{\rho_{b}}$$

so that eq 37 becomes

39
$$h\left(r\right)=h\left(r\right)-\ln\left(h\left(r\right)+1\right)-\beta \phi \left(r\right)-\beta H_{c}\left[\rho_{b}g\left(r\right)\right]+\rho_{b}\int dr^{\prime }h\left(r^{\prime }\right)c_{c}^{\left(2\right)}\left(|r-r^{\prime }|\right)-\rho_{b}\int dr^{\prime }h\left(r^{\prime }\right)\beta \phi_{\mathrm{sh}}\left(|r-r^{\prime }|\right)$$

If *F*_c_[ρ] were exact, then −β*H*_c_[ρ_b_ *g*(*r*)] would be the bridge-function *B*_c_(*r*) of the reference hard disk fluid.^22^ Thus, comparing the first four terms on the right-hand side of eq 39 with the right-hand side of eq 30, we see that these four terms together correspond to an approximation for *c* ^(2)^(*r*) that is neither the exact result (eq 30), nor the RPA approximation (eq 31). This close similarity to the exact expression in eq 30, with the only difference being the approximation effectively made for the bridge function *B*(*r*), formed the basis of the arguments in ref 22 that mean-field DFT is often better than one might expect. It is also one reason why the test-particle route to *g*(*r*) is generally expected to be superior to the OZ route.

## Appendix B

The DFT calculations were performed using a code written by the authors that is available online,^53^ using distributed memory parallelization of the FFT via the mpi4py-fft package.^54^ The RDFs are obtained using Picard iteration; for details on the numerical implementation we refer to previous work using the same software.^3^ The test-particle located at (*x* = 0, *y* = 0) is included via the shoulder part of the potential in eq 14, treated explicitly as external potential term in the minimized free energy, while the hard core repulsion is enforced by setting the density within the core ρ­(*r* < σ) ≡ 0, at every iteration step. The calculations are carried out in square boxes of size *L*_*x*_ = *L*_*y*_ = 50σ, discretized on *N*_*x*_ = *N*_*y*_ = 2048 grid points using periodic boundary conditions. The resulting density profiles are then angular-averaged according to eq 29 using 500 evenly spaced radial bins. To verify that neither the resolution nor the boundary conditions are affecting the described features of *g*(*r*), we performed DFT calculations for λ = 4.9 at η = 0.4 varying both box size and number of grid points and found no significant effect of both computational parameters on the resulting *g*(*r*). A calculation was considered converged, if the cumulative squared error between the densities of consecutive steps in the Picard iteration fell below 10^–13^.

The *g*(*r*) via the OZ route are calculated from the structure factor *S*(*k*) = [1 – ρ *^b* ^(2)^(*k*)]^−1^ (see eq 16) by numerical integration of the inverse Hankel transform

40
$$g\left(r\right)=1+\frac{1}{2\pi \rho \mathrm{b̂}}\int_{0}^{\infty }\left(S\left(k\right)-1\right)kJ_{0}\left(\mathrm{kr}\right)dk$$

The radius in Fourier space *k* ∈ (0.025,250) was discretized uniformly with grid spacing d*k* = 0.025. The numerical integration of the above integral was then performed for the uniformly spaced real space radii $r\in \left(\frac{2\pi }{250},\frac{2\pi }{dk}=2\pi \cdot 40\right)$ .
